# Variation of Nanostructures, Molecular Interactions, and Anisotropic Elastic Moduli of Lignocellulosic Cell Walls with Moisture

**DOI:** 10.1038/s41598-017-02288-w

**Published:** 2017-05-17

**Authors:** S. Youssefian, J. E. Jakes, N. Rahbar

**Affiliations:** 1 Department of Mechanical Engineering, Worcester Polytechnic Institute, Worcester, MA 01609 USA; 2 Forest Biopolymers Science and Engineering, USDA Forest Service, Forest Products Laboratory, Madison, WI 53726 USA; 3 Department of Civil and Environmental Engineering, Worcester Polytechnic Institute, Worcester, MA 01609 USA

## Abstract

A combination of experimental, theoretical and numerical studies is used to investigate the variation of elastic moduli of lignocellulosic (bamboo) fiber cell walls with moisture content (MC). Our Nanoindentation results show that the longitudinal elastic modulus initially increased to a maximum value at about 3% MC and then decreased linearly with increasing MC. In contrast, the transverse moduli decrease linearly with MC. We showed that amorphous materials in cell walls have key roles in the variation of elastic modulus with increasing MC. Elastic modulus of lignin, calculated by molecular dynamics simulations, increases initially with increasing MC, and then decreases. In contrast, elastic modulus of hemicellulose decreases constantly with MC. Below 10% MC, water molecules tend to break hydrogen bonds between polymer chains and form new hydrogen bond bridges between the polymer chains, while above 10% MC, water molecules aggregate together and create nano-droplets inside the materials. During the process of bridging, the fractional free volume of lignin decreases. The free volume reduction along with formation of hydrogen bond bridges causes a growth in elastic modulus of lignin at low MC. The constant increase of hemicellulose free volume, however, causes the aggregation of voids in the system and diminution of elastic properties.

## Introduction

Bamboo fiber is a unique material with outstanding strength and rigidity to weight ratios. Considering its low density (1.05 g/cc) and relatively high tensile strength and elastic modulus (800 MPa and 50 GPa, respectively), bamboo fiber strength and rigidity to weight ratios are higher than that of many metal alloys and ceramics^1–4^. The remarkable mechanical properties of bamboo fibers come from its natural composite cell wall structure in which cellulose microfibrils are embedded in a matrix of hemicelluloses, lignin, and a crosslinked structure of hemicellulose and lignin termed the lignin carbohydrate complex (LCC). Although the structure of bamboo fiber cell wall is complicated and not entirely understood, it is known that the cellulose microfibrils with ordered and amorphous regions are oriented approximately parallel to the fiber longitudinal axis. Ordered and amorphous regions are formed due to organized and random hydrogen bonds between the hydroxyl groups of aldehyde sugar molecules, respectively. We have previously shown that the molecular origin of the longitudinal rigidity of bamboo fibers comes from the carbon-carbon and carbon-oxygen covalent bonds in the ordered region of cellulose microfibrils^5^. In the cell wall matrix of bamboo fiber, hemicellulose exhibits larger elastic modulus while lignin shows greater tendency to adhere to ordered cellulose microfibrils. Consequently, the role of hemicellulose is found to be enhancing the transverse elasticity of the matrix by forming dense hydrogen bond networks while the role of lignin is found to be providing the strength of bamboo fibers by creating strong van der Waals forces between cellulose microfibrils and the matrix.

Another component of the cell walls is water, which is known to have profound effects on the mechanical properties of wood. At the cell wall level, nanoindentation studies have shown that the longitudinal elastic modulus of S2 secondary layers in wood cell wall decreases with increasing moisture content (MC)^6, 7^. The results in these studies support the assumption that the moisture- sensitive matrix is responsible for the change in modulus of elasticity. In order to fully understand the plasticization effect of moisture on the individual wood cell wall constituents, Cousins measured the elastic moduli of extracted lignin and hemicellulose^8–10^. He observed a small increase in elastic modulus of lignin from MC of 0.0% to 3.6%, and linear regression at the higher MC. For hemicellulose, up to 10% MC the loss of modulus is small, but from 10% to nearly 70% MC the elastic modulus decreases by nearly three orders of magnitude. In another attempt, to understand the mechanism of water interactions with wood cell walls Kulasinski *et al*. employed molecular dynamics simulations and showed that increasing MC above 10%, decreases the stiffness of amorphous cellulose, noticeably^11^. Their results suggest that the moisture is adsorbed in hemicellulose and the interfaces of cellulose/hemicellulose, while no sorption occurs in the ordered cellulose^12^.

In spite of the significance of these studies, the effective factors in variation of the anisotropic mechanical properties of cell walls with MC and the mechanism behind the water aggregation in cell wall are not fully understood. In this paper, with a combination of experimental, theoretical and numerical studies, we shed light on the influence of MC on the molecular interactions, nanostructure and mechanical properties of bamboo fibers. In particular, we untangle the complex roles of each constituent of bamboo fiber in response to water permeation by revealing the variation of their nanostructures and molecular interactions. The results of this study can also be used to gain new insights into the nanoscale organization of the polymeric constituents and the fiber wall structure-property relationships.

## Results

### Elastic Modulus Variation with Moisture Content

In order to achieve this goal, nanoindentation was used to investigate the anisotropic moisture-dependent elastic moduli of bamboo fiber cell walls. Surfaces in the transverse and longitudinal orientations were prepared for experiments using a Sorvall MT-2 ultramicrotome equipped with a diamond knife following procedures previously developed for wood cell walls^13, 14^. Fiber wall elastic moduli were assessed using a Hysitron (Minneapolis, Minnesota, USA) TriboIndente equipped with a Berkovich probe following the procedures in Jakes *et al*.^14^. In brief, load-control multi-load nanoindents were placed in the center of fiber cell walls and the resulting load-depth traces were corrected for edge effects and specimen-scale flexing using the structural compliance method^13^. Then, the Oliver-Pharr method^15, 16^ was used to calculate elastic modulus from the corrected load-depth traces. The relative humidity (RH) inside of the nanoindentation enclosure was controlled with an InstruQuest (Coconut Creek, Florida, USA) HumiSysTM HF RH generator. Specimens were conditioned in absorption inside the enclosure for at least 36 hours at room temperature (24–26 °C) under each RH and the RH was maintained during the experiments. On the longitudinal plane 6–7 multi-load nanoindents were performed at each RH along a single fiber wall. All nanoindents performed on the transverse plane were placed on fibers from a single bundle of vascular fibers and 11–18 nanoindents were placed across 5–8 fibers at each RH.

**Figure 1:**
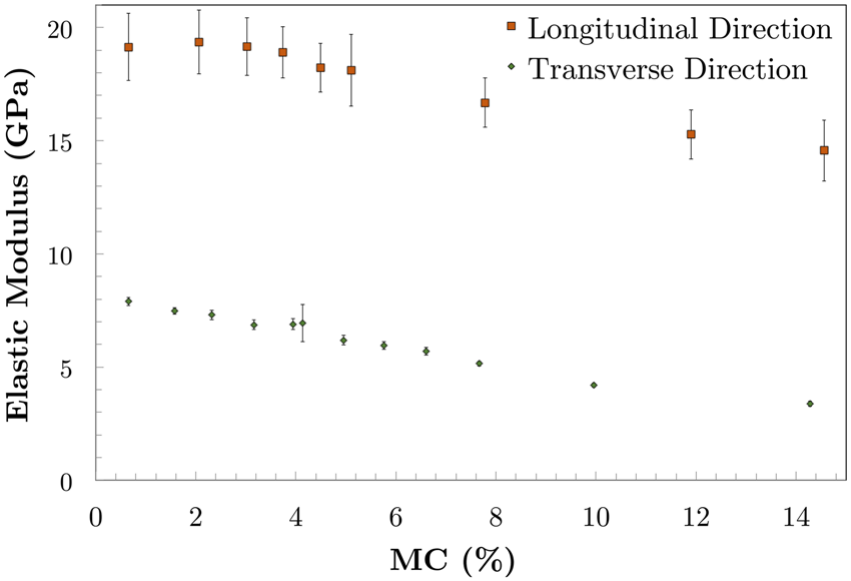
Experimental longitudinal and transverse nanoindentation elastic moduli of bamboo fiber walls as a function of moisture content (MC). The longitudinal elastic moduli were assessed from the surface prepared in the transverse plane in which the cellulose microfibrils were oriented approximately parallel with indentation direction. Similarly, the transverse elastic moduli were assessed from the surface prepared in the longitudinal plane in which the cellulose microfibrils were oriented approximately perpendicular to the indentation direction.

Nanoindentations of longitudinal and transverse elastic moduli from experiments are shown as a function of MC in Fig. 1. The stiff cellulose microfibrils, which are oriented close to parallel and perpendicular in the longitudinal and transverse elastic moduli results, respectively, are the most dominant structural feature that affects the anisotropic elastic moduli in Berkovich nanoindentation of cell walls^17^. The nanoindentation elastic moduli and the differences between the transverse and longitudinal surfaces agree well with previous work performed under ambient lab conditions^17^. These results show that the longitudinal elastic modulus remained considerably higher over the range of MCs tested. The longitudinal elastic modulus is strongly influenced by longitudinal modulus of cellulose microfibrils, which provides higher modulus of elasticity due to its partly crystalline structure.

Distinguishable differences were also observed in the shapes of the elastic modulus curves with MC and these differences are of most interest in the paper to gain new insights into the fiber wall structure-property relationships. The transverse elastic modulus continuously decreases almost linearly with increasing moisture content at the rate of 0.35 GPa per 1% moisture content from 7.9 GPa to 2.6 GPa. In contrast, the longitudinal elastic modulus initially increases from an average value of 19.1 GPa at 0.5% moisture content to maximum of 19.4 GPa at approximately 3% MC, and then decreases to 14.6 GPa at 14.5% with a rate of 0.4 GPa per 1% moisture content. A slight increase or constant elastic modulus at low MCs has also been observed in bulk wood elastic moduli parallel to the grain, but elastic moduli perpendicular to the grain decrease consistently with increasing MC even at low MCs^18^.

### Principle Factors that Control the Elastic Modulus Variation

To elucidate the mechanisms that control the nonlinear response of the longitudinal elastic modulus, we need to reveal the factors that dictate the elastic modulus of this material. The principle factors of the elastic behavior are known to stem from the changes in the free energy density of a material (f = F/V, where F is the free energy and V is the volume)^19^. Free energy density is composed of potential energy density of the whole system (u) and its entropy density (s):

1 $$f=u-Ts$$

where T is the temperature. Modulus of elasticity (*E*) can be calculated from the second derivative of the free energy density with respect to strain,

2 $$E=\frac{{\partial }^{2}f}{\partial {\varepsilon }^{2}}$$

This equation suggests that the elasticity of a material is strongly influence by the contributors to the free energy of a unit volume of the material. At moderate temperatures and small deformation the contribution of the entropy is not significant. Therefore, the entropic term can be neglected and the elastic behavior of a material can be intimately linked to the potential energies density of the system.

3 $$E=\frac{{\partial }^{2}U}{\partial {\varepsilon }^{2}}$$

The potential energy density is the resultant of the interactions between atoms of a unit volume of materials. The potential energies can be considered as a spring that connects the atoms together. With a simple calculation, the ensued stiffness (*K*) can be estimated from the generic function for defining the behavior of interaction energy between two atoms (Lennard-Jones potential energy function).

4 $${U}_{LJ}=\in [{(\frac{{r}_{m}}{r})}^{n}-2{(\frac{{r}_{m}}{r})}^{m}]$$

where *r* is the distance between the two atoms, ϵ is the depth of the potential well and *r*_*m*_ is the equilibrium distance. The stiffness around the equilibrium distance of two atoms can be expressed from the second derivative of the potential energy, $K={[\frac{{\partial }^{2}{U}_{LJ}}{\partial {r}^{2}}]}_{r={r}_{m}}$.

5 $$K=\frac{[n(n-1)-2m(m-1)]{\epsilon }}{{r}_{m}^{2}}$$

**Figure 2:**
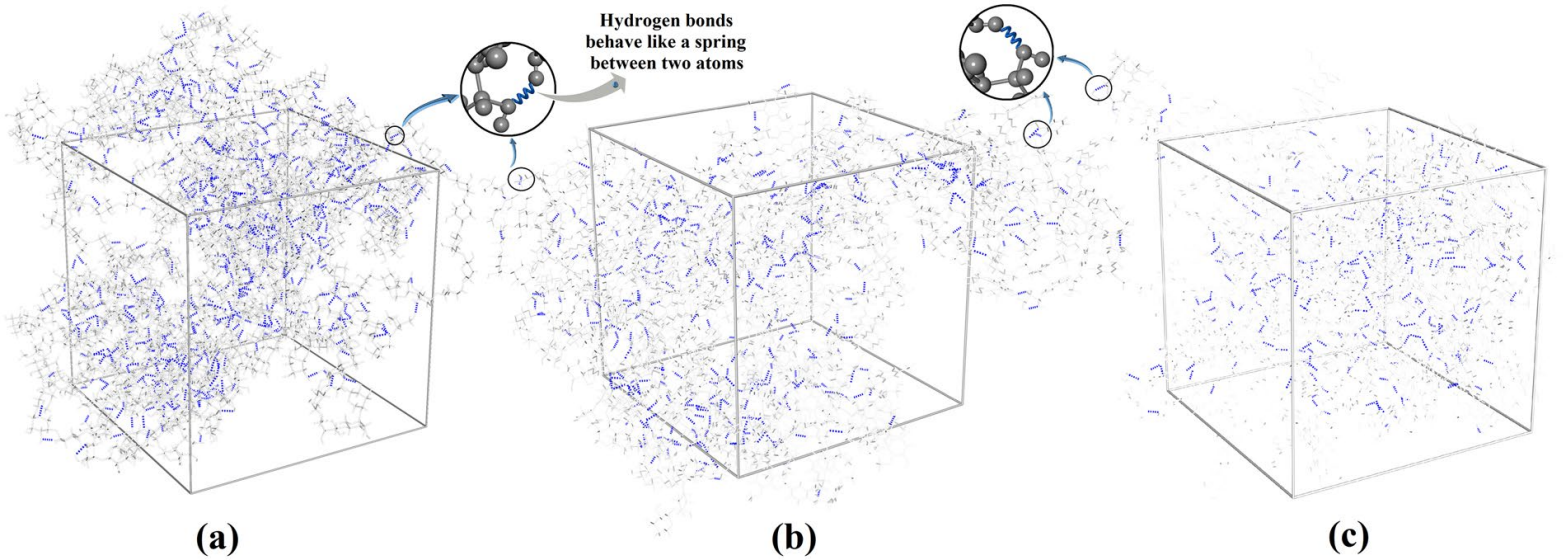
Hydrogen bond distributions of (**a**) Hemicellulose (**b**) LCC (**c**) Lignin. Hemicellulose with larger number of hydroxyl groups forms denser hydrogen bond network than lignin. LCC consists of hemicellulose and lignin molecular structure and has an intermediate hydrogen bond network between hemicellulos and lignin. These hydrogen bonds, which act like a spring between atoms, constrain their movement and dictate the elastic behavior of these materials.

This simplified definition of the stiffness of the potential energy indicates that strong short-range potential energies (larger ϵ and smaller *r*_*m*_) connect atoms with stiffer interactions than weak long-range potential energies. As a result, covalent bonds connect atoms with higher stiffness than hydrogen bonds and hydrogen bonds with higher stiffness than other non-bonded potential energies. In materials such as ordered cellulose in which external loads predominantly struggle against C-C and C-O, covalent bond potential energies dominate the elasticity and escalate the elastic modulus to relatively high value (E_X_ = 11–57 GPa, E_Y_ = 50–57 GPa and E_Z_ = 110–200 GPa^16–19^. For amorphous materials such as the fiber cell wall matrix constituents covalent bonds do not play significant roles whereas hydrogen bonds primarily influence the elasticity, causing relatively low modulus elasticity of 6.93 GPa^5^. Since water molecules with capabilities of interfering with hydrogen bond network are more likely to alter the elastic response of the matrix than the ordered regions of cellulose microfibrils, we focus our study on the influence of water molecules on hydrogen bond energies and nanostructures of bamboo fiber matrix and its constituents. Figure 2 illustrates the distributions of hydrogen bonds for hemicellulose, lignin and LCC (the matrix). It shows that lignin and LCC have lower number of hydroxyl groups and are less hydrophilic compared to hemicellulose^20–24^. Therefore, the influence of water molecules on the hydrogen bond network of lignin, LCC and hemicellulose should be different. In order to explore the different responses of the matrix constitutes to absorbed moisture, atomistic simulations have been used as a powerful tool to elucidate the variation of the molecular interactions and nanostructures.

### Underlying Mechanisms of Elastic Modulus Variation

**Figure 3:**
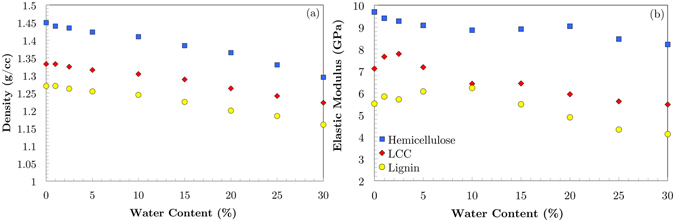
Variation of density and elastic modulus of hemicellulose, lignin and LCC with increasing MC. Hemicellulose density constantly decreases with growing MC whereas LCC and lignin are resistant to the variation of density at very low MC. Hemicellulose elastic modulus constantly decreases with increasing MC whereas LCC and lignin elastic moduli increase at low MC and then decrease.

Figure 3 elucidates the variations of the density and modulus of elasticity of hemicellulose, lignin and LCC with MC. Hemicellulose with higher density than lignin and LCC exhibits more sensitivity to water molecules. Introducing a small amount of water (less than 2.5%) into dry LCC and lignin does not change their densities significantly whereas hemicellulose density decreases notably. As the water molecules continue permeating into these polymers, their densities decrease almost linearly. The elastic modulus of hemicellulose drops almost linearly with increasing water content whereas lignin and LCC elastic moduli increase at low water content (less than 10%) and decreases afterwards. The peaks in elastic moduli are at 2.5% and 10% MC for LCC and lignin, respectively. When water molecules permeate into the polymer, they enhance the elastic modulus of the material. Once the number of water molecules inside the material reaches a certain level, the effect of water molecules becomes regressive and the modulus of elasticity starts to decrease.

Comparison between the shapes of the moisture-dependent elastic moduli from the nanoindentation experiments (Fig. 1) and simulation results (Fig. 3b) reveals interesting similarities. The transverse nanoindentation elastic moduli have a similar shape to the hemicellulose simulations and the longitudinal nanoindentation elastic moduli have similar shape to the LCC and lignin simulations. This suggests that the nanostructure of fiber is organized in such a way that when compressed in the direction perpendicular to the cellulose microfibrils, hemicellulose, or possibly the similarly structured amorphous cellulose, has more influence on the elastic response than lignin. Conversely, in the direction parallel to the cellulose microfibrils the lignin has a larger effect than the hemicellulose on the elastic response.

**Figure 4:**
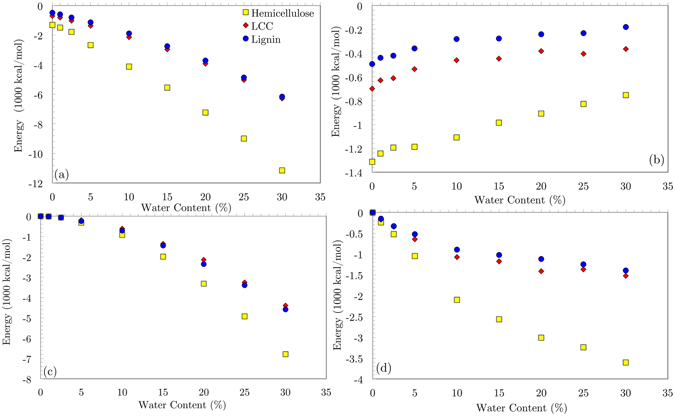
Hemicellulose, lignin and LCC variation of hydrogen bond energies (**a**) between all particles (**b**) between just polymer chains (**c**) between just water molecules (**d**) between polymer chains and water molecules. At lower than 10% MC, while hydrogen bond between polymer chains are broken by water molecules, they are being replaced by hydrogen bonds between water molecules and polymer chains. At more than 10% MC, water molecules tend to form hydrogen bonds with other water molecules and create nano-droplets in the systems.

The intermolecular energies between particles in a material play an important role in determining its molecular structure that intimately dictates the physical properties such as density and elastic modulus. Since hydrogen bond interactions are both short range and angle-dependent, their impacts on structuring of polymers are significant. In hemicellulose and lignin, due to their high number of hydroxyl groups, the consequences of hydrogen bond energies are amplified, especially when interacting with water molecules. Studying variation of hydrogen bonds with water content allows us to capture important physics of water-polymer interactions with a minimal need for experimental inputs. Hence, we start with investigating the total hydrogen bond energies of hemicellulose, LCC and lignin in different MC (Fig. 4a). All three materials exhibit almost the same progressive trend of total hydrogen bond energies when the water content increases. Nevertheless, hemicellulose shows higher total hydrogen bond energy than LCC and lignin due to the larger number of hydroxyl groups.

In an effort to investigate the conformational change of the hemicellulose, LCC and lignin with increasing MC, the hydrogen bond energies between merely polymer chains are calculated and shown in Fig. 4b. At low MC, the diminution of hydrogen bond energies indicates that hydrogen bonds are being broken by water molecules. Higher than 10% MC, the lignin and LCC curves go to almost a plateau, indicating a saturation point for hydrogen bond rupture process. The hydrogen bond energy between water molecules are presented in Fig. 4c. Higher than 10% MC, the growth of hydrogen bond energies between water molecules increases significantly. This suggests that water molecules tend to create hydrogen bond with other water molecules and aggregate at high water content. Figure 4d presents the hydrogen bond interactions between polymer chains and water molecules. The salient growing trend of this graph below 10% MC shows that the water molecules tend to form hydrogen bonds with polymer chains at low MC.

Taken all together, the results on Fig. 4 suggest that at low MC the hydrogen bonds between the polymer chains and water molecules seem to be more favorable than water-water hydrogen bond interactions. At higher MC, however, water molecules tend to make hydrogen bond with other water molecules and form nano-droplets inside the materials.

**Figure 5:**
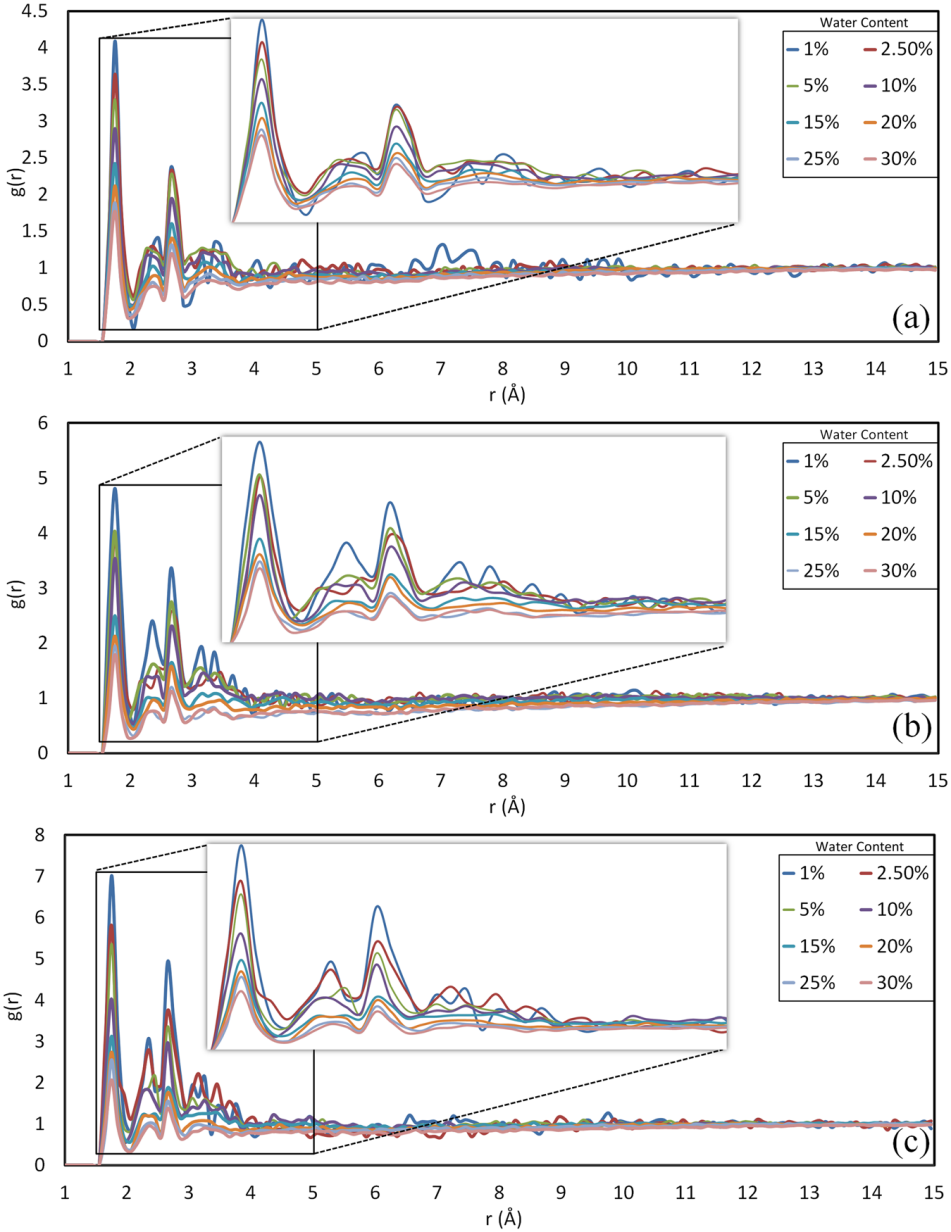
Radial distribution functions between water molecules and hydroxyl groups of (**a**) Hemicellulose (**b**) LCC (**c**) Lignin. First peaks at 1.75 Å indicate strong and short-range hydrogen bonds between water molecules and polymer chains. Higher peaks of lignin and LCC at 1.75 Å suggest that larger portion of water molecules interacts with lignin and LCC chains than that of hemicellulose.

In order to analyze the evolution of hydrogen bond characteristics with increasing water molecules, Radial Distribution Functions (RDFs) between water molecules and hydroxyl groups of hemicellulose, LCC and lignin were calculated (Fig. 5). RDF is a structural characterization parameter of amorphous molecules that provides the basic information about short-range order and the nature of atomic interactions. RDF gives the possibility of finding a particle at a certain distance from the reference particle. The observed variations in dihedral conformation and internal structural variables are the results of the dynamics of non-bonded interactions like hydrogen bonding, electrostatic and van der Waals interactions. The first three distinct peaks on Fig. 5 are consequences of hydrogen bonding. The highest peak appearing at 1.75 Å is associated with the donor – acceptor distance of hydrogen bonds. This shows a short-range strong hydrogen bond between water molecules and hydroxyl groups on polymer chains. The second peak at 2.45 Å appears due to distance between donor and hydrogen on the acceptor side. The third peak at 2.65 Å represents the distance between water molecule oxygen and the oxygen on the hydroxyl groups. The next successive peaks are indications of long-range electrostatic interactions between water molecules and hydroxyl groups. The diminution of the peak heights indicates that when the MC increases, the fraction of water molecules in contact with the polymers decreases. In other words, as the MC increases, water molecules tend to form hydrogen bonds with other water molecules rather than the polymer chains. Nonetheless, there is a significant difference between hemicellulose and lignin RDFs. Unlike hemicellulose, lignin peaks drops suddenly after 10% water content. This suggests that lignin nanostructure is saturated after 10% MC. LCC also follows the lignin behavior.

The other difference between lignin and hemicellulose response to water molecules is the height of the peaks. The higher peaks on lignin diagram indicate higher probability of finding the water molecules around the reaction site on lignin chains than that of hemicellulose. Therefore, lignin tends to interact with a larger fraction of introduced water molecules than hemicellulose. This is due to the position of functional groups adjacent to the hydroxyl groups. The lignin hydroxyl groups are mostly parts of well-spread hydroxymethyl groups that are extended out from the main chains. The hemicellulose hydroxyl groups, however, are parts of xylose groups, where two hydroxyl groups attach directly to the main chains and are localized on pyranose rings. Lignin hydroxyl groups are more exposed to and more accessible by water molecules than those of hemicellulose hydroxyl groups. Hence, although the hemicellulose structure contains more hydroxyl groups, lignin hydroxyl groups are more efficient in making hydrogen bonds.

Studying the fractional free volumes (FFVs) of a material can bring a deeper insight into understanding the nanostructure and the transport behavior of nonporous amorphous polymers. FFV can be defined as the ratio of the specific free volume (υ_f_) to the total specific volume (υ_T_). υ_f_ can be calculated by subtracting the occupied volume (υ_OC_) from the total volume, υ_f _ = υ_T_ − υ_OC_. Therefore, the FFV can be calculated from,

6 $$FFV=1-\frac{{{\rm{\upsilon }}}_{{\rm{OC}}}}{{{\rm{\upsilon }}}_{{\rm{T}}}}$$

**Figure 6:**
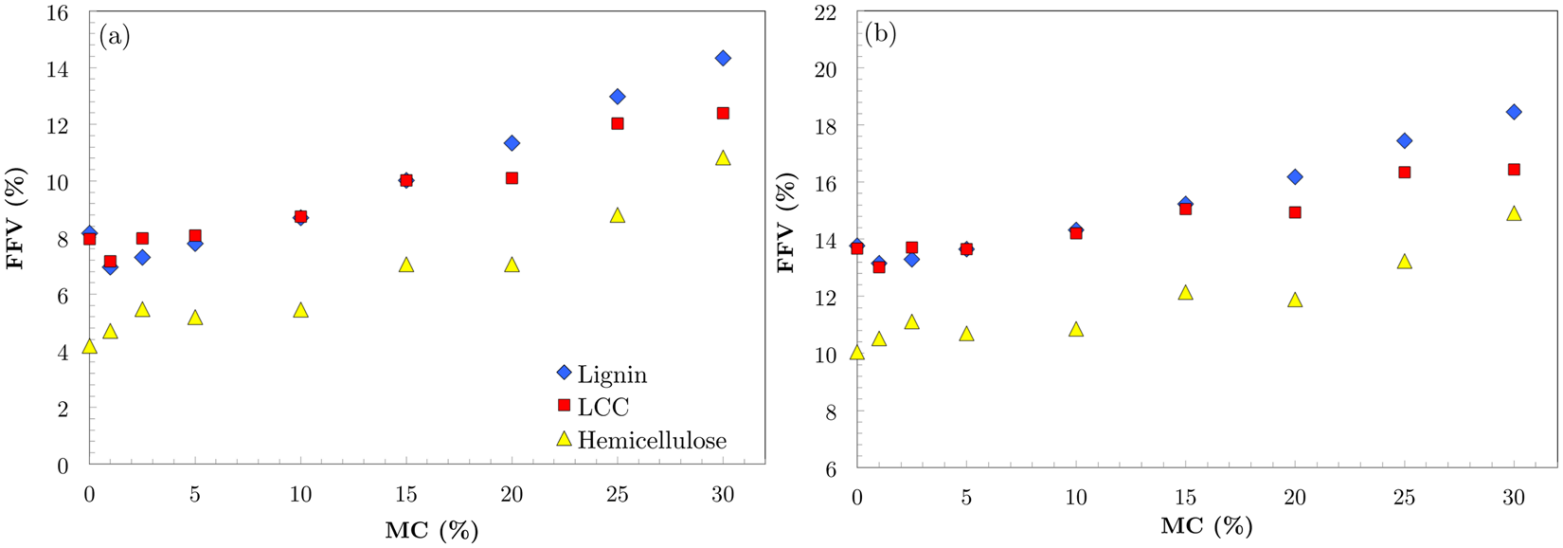
Variation of fractional free volume (FFV) of hemicellulose, lignin and LCC calculated by (**a**) VABC (**b**) Connolly methods. Lignin and LCC have more free space for water molecules to fit in. As the water molecules are introduced to the system, they fit into the free volume between polymer chains and reduce FFV at low MC. Hemicellulose has denser structure. Adding water molecules to this system, expands the volume and increases the portion of free volume, even at low MC.

Figure 6 shows the variation of FFV with MC for lignin, LCC and hemicellulose, calculated by VABC and Connolly methods (Fig. 5a and b, respectively). Although at higher MCs, VABC estimates FFV at higher values than Connolly, both approaches show the same trend for all three materials. The lignin FFV in dry condition is about 14%. Introducing water into lignin decreases the free volume down to 13% at 2.5% MC, followed by a linear increase to ~20% around 30% MC. Hemicellulose, however, exhibits different response to increasing water molecules. Dry hemicellulose has an FFV around 10%, indicating a denser structure than lignin and LCC. Introducing water into the hemicellulose structure increases the portion of free volume to 12% rapidly followed by a linear increase up to ~17% at 30% MC. The FFV value of LCC drops to 13% once the water is introduced into the system and then increases linearly with growing MC. This behavior seems to be influenced more by lignin than hemicellulose.

The different trends of variation of lignin and hemicellulose FFV with increasing MC indicate different influence of water molecules on the nanostructure of these materials. Hemicellulose with a dense hydrogen bond network has denser molecular structure than lignin. Upon the permeation of water molecules into hemicellulose molecular structure, free volume of this material increases because the system expands rapidly to create enough space for water molecules to fit in. Lignin with lower number of hydroxyl groups forms a different molecular structure with more free space available for water molecules to fit to. At low MCs, water molecules take spaces between the lignin polymer chains and decrease the free volume of this material. After 10% MC, however, the nano-droplets are formed in lignin and free volume linearly increases with increasing MC.

## Discussion

In this study, we have employed a combination of experimental, theoretical and numerical approaches to study the variation of anisotropic elastic moduli of bamboo fibers with increasing moisture content (MC). Nanoindentation was used to experimentally assess the longitudinal and transverse elastic moduli of bamboo fiber cell walls from 0 to 15% MC. Since the stiff cellulose microfibrils are oriented closely to parallel with longitudinal direction, longitudinal elastic moduli were consistently higher than the transverse elastic moduli over the entire MC range. However, the trends with increasing MC were different. In the longitudinal modulus, the elastic moduli initially increased to a maximum at approximately 3% MC and then decreased linearly with increasing MC. In contrast, throughout the MC range tested the transverse moduli decreased linearly with MC. In order to explain the mechanisms controlling the nonlinear trends of elastic moduli, the principle factors that dictate the elastic behavior of bamboo fibers were elucidated. We theoretically demonstrated that the molecular interaction energies that govern the elastic behavior of ordered cellulose are covalent potential energies whereas for amorphous constituents of bamboo fibers hydrogen bond energies govern the elastic modulus. Since water molecules are capable of interfering with hydrogen bond networks, the variation of hydrogen bond energies and nanostructures of amorphous materials such as lignin, hemicellulose and LCC have the major roles in dictating the elastic modulus variation with MC. Molecular modeling techniques have been used to calculate properties, hydrogen bond interactions and nanostructures of hemicellulose, lignin and LCC, at different MCs. The results suggest that lignin elastic modulus initially increases with increasing MC and then decreased whereas elastic modulus of hemicellulose decreases constantly with MC. This trend is in good agreement with the trend observed by previous experiments on lignin and hemicelluloses extracted from wood cell walls (Cousins, 1976; Cousins, 1977; Cousins, 1978). Comparing numerical and experimental results indicates that between the fiber matrix constituents hemicellulose has a larger influence on the moisture-dependent transverse elastic modulus of bamboo fiber whereas lignin has larger impact on longitudinal elastic modulus. Consequently, these polymers would need to be targeted to modify the elastic moduli of bamboo fiber in corresponding directions. We also found two distinct mechanisms governing the aggregation of water molecules in lignin, LCC and hemicellulose. At low MC, water molecules prefer to take spaces between the polymer chains and form hydrogen bond bridges between them. At high MC, water molecules tend to make hydrogen bond with other water molecules and form nano-droplets inside the materials. These two mechanisms have different effects on the nanostructures of lignin and hemicellulose. At low MC, while water molecules are forming hydrogen bond bridges, the free volume in lignin decreases. In contrast, water molecules expand relatively dense nanostructure of hemicellulose and increase the free volume. Consequently, at low MC, the elastic modulus of lignin slightly increases whereas hemicellulose elastic modulus decreases. At high MC, the formation of nano-droplets in both lignin and hemicellulose nanostructures develops voids in the materials and results in the reduction of elastic moduli. LCC seems to adopt lignin response to variation of MC. These results may also provide new insights into how engineering the molecular scale structures of cell wall polymers may lead to the ability to independently control the elastic moduli of bamboo fiber in different directions.

## Materials and Methods

### Molecular Structure of Amorphous Bamboo Cell Wall

Lignin is a natural phenolic macromolecule that mainly presents in the plant secondary cell wall. It is made up of three main phenylpropanoid sub-units, namely p-hydroxyphenyl (H-type), guaiacyl (G-type) and syringyl (S-type) units^25^. In this study, a structural model with 28 subunits of lignin proposed by Sakakibara (1980) has been used^26^. In this model, the value of the structural units and the number of protons per C9 structural units are close to that of spruce milled wood lignin reported by other researchers^26^. Hemicelluloses are a heterogeneous group of polysaccharides that unlike the cellulose, frequently have side chain groups. They are essentially amorphous with little strength^27^. Bamboo hemicellulose has been shown to be a xylan and further characterized as 4-O-methy1-D-glucurono- arabino-xylan^28^. Hemicellulose molecules bond to lignin by a variety of different chemical bonds, however, most of the evidence refers to ether and ester bonds. Jeffries proposed structures for ester and ether linkages for lignin/uronic acid and lignin/arabinoxylan groups, respectively^29^. These linkage models have been used to create the bonds between lignin and hemicellulose in the crosslinked LCC network. More details on the modelling of these materials can be found in ref. 5.

### Atomistic simulations

COMPASS (cff91 ver. 2.644) was chosen as a proper force field for atomistic simulations. In COMPASS, the non-bonded energies include van der Waals and electrostatic energies, with hydrogen bonds being a natural consequence of electrostatic energies. To study the hydrogen bond energies between particles the following criteria have been used:

1. The maximum distance between the hydrogen and the acceptor atom for which hydrogen bonding is possible is 2.5 Å.
2. The minimum angle between the donor, hydrogen and acceptor atoms in degrees for which hydrogen bonding is possible was chosen as 120°.

We have used a CHARMM-like hydrogen bonding potential such as Equation (7) to calculate the hydrogen bond energies between particles,

7 $${E}_{hb}={D}_{hb}[5{(\frac{{r}_{hb}}{{r}_{DA}})}^{12}-6{(\frac{{r}_{hb}}{{r}_{DA}})}^{10}]{\rm{c}}{\rm{o}}{{\rm{s}}}^{4}({\theta }_{DHA})$$

where θ_DHA_ is the bond angle between hydrogen donor (D) and the hydrogen (H) and the hydrogen acceptor (A). RDA is the distance between the donor and acceptor. The values of D_hb_ and r_hb_ were adopted from the literature^30^.

To study the nanostructures of the models, Radial Distribution Function (RDF) was used. RDF gives a measure of the probability of finding an atom, within a spherical shell of infinitesimal thickness at a distance, r, from the reference atom. To calculate elastic modulus, the periodic structures were expanded along each direction to the maximum strain amplitude of 0.01 in 10 steps. In each step the stresses were obtained from virial stress expression which is commonly used to relate the computed stress in molecular dynamics to continuum stresses.

To calculate, fractional free volume of linin, hemicellulose and LCC, Bondi has been used to compute the occupied volumes by the particles. Bondi estimated the occupied volume from the packing density of molecular crystals at 0 K to be approximately 1.3 times the van der Waals volume (υ_W_) of the repeat unit of the polymer which can be reliably estimated from group contributions^31, 32^. Here, we have used a method developed by Zhao *et al*. for fast calculation of van der Waals volume as a sum of atomic and bond contributions^33^. In this method, which is termed as Atomic and Bond Contributions of van der Waals volume (VABC), the only information needed for calculating the υ_W_ is the atomic contributions and the number of atoms, bonds, and rings as follows,

8 $${{\rm{\upsilon }}}_{{\rm{W}}}=14.71{N}_{O}+20.58{N}_{C}+7.24{N}_{H}-5.92{N}_{Bonds}-14.7{N}_{ARings}-3.8{N}_{NARings}$$

where, N_O_, N_C_, N_H_, N_Bonds_, N_ARings_ and N_NARings_ are numbers of oxygen, carbon, hydrogen, bonds, aromatic rings and nonaromatic rings, respectively. The oxygen, carbon and hydrogen atoms have been assumed to be represented by hard spheres with a radius derived from the van der Waals radius of respective atoms (r_C_ = 1.7 Å, r_H_ = 1.2 Å, r_O_ = 1.55 Å)^34^.

The number of bonds can be simply calculated by,

9 $${N}_{Bonds}=N-1+{{\rm{N}}}_{{\rm{ARings}}}+{{\rm{N}}}_{{\rm{NARings}}}$$

where N (the total number of atoms) is the summation of all atoms in the system. Therefore, Equation (8) can be modified to,

10 $${{\rm{\upsilon }}}_{{\rm{W}}}=8.79{N}_{O}+14.93{N}_{C}+1.32{N}_{H}-20.62{N}_{ARings}-9.72{N}_{NARings}+5.92$$

Although VABC approach has proven to provide very useful correlative and even predictive capabilities, its capabilities have been proven accurately merely for organic compounds. Since in our models water molecules have a major contribution in the expansion of the nanostructure, we have also employed more accurate, yet more complicated theory for calculating the occupied volume by Connolly^35^.

Connolly method analytically calculates the solvent-excluded volume of a molecule by a direct determination of the volume enclosed by the solvent accessible surface. The molecule is modeled as a static collection of hard spheres (with van der Waals radii) which completely exclude a spherical probe representing a solvent molecule. As the probe rolls over the van der Waals surface, it creates a Connolly surface that is a combination of three kinds of surface: convex, saddle, and concave. The total solvent-excluded volume of the molecule is the summation of all these surface pieces. Here, the volume of each polymer was calculated with Connolly method using a probe radius of 0 Å which returns the van der Waals volume of carbohydrates.

### Nanoindentation Experiment

In nanoindentation, a carefully shaped diamond probe is pressed into a material following a prescribed loading function. From the resulting load-depth trace, mechanical properties, most often elastic modulus can be assessed. Nanoindentation requires ultrasmooth surfaces. To avoid any potential effects of epoxy embedment, nanoindentation surfaces in s bamboo (solid sample labeled “high density of fibers” on bag) was prepared without embedment. Transverse and longitudinal surfaces were prepared in bamboo. A Hysitron (Minneapolis, Minnesota, USA) TriboIndenter® equipped with a Berkovich probe was used. The machine compliance, probe area function, and tip roundness effects were determined from a series of 80 load control nanoindents in a fused silica standard. The calibration load function consisted of a 4-s loading segment, 4-s hold at maximum load P0, 2-s unload to 40% P0, 60-s hold at 40% P0 ¬to assess thermal drift, and a 1-s final unload. Each nanoindent was preceded by a 20-nm liftoff to assure accurate surface detection. The contact stiffness was assessed by fitting the Oliver–Pharr power law function^15^.

In bamboo, nanoindents were placed in S2 secondary cell wall layers in neighboring fiber cells in the transverse surface and along one of two S2 secondary cell wall layers in the longitudinal surface. The relative humidity (RH) inside of the nanoindentation enclosure was controlled with an InstruQuest (Coconut Creek, Florida, USA) HumiSys^TM^ HF RH generator. Specimens were conditioned inside the enclosure for at least 36 hours at each condition and the RH was maintained during the experiments. Experiments were performed in absorption at 1, 12, 17, 23, 30, 39, 56, and 77% RH.
